# ZnO Nanostructure-Based Intracellular Sensor

**DOI:** 10.3390/s150511787

**Published:** 2015-05-21

**Authors:** Muhammad H. Asif, Bengt Danielsson, Magnus Willander

**Affiliations:** 1 Department of Physics, COMSATS Institute of Information Technology, Lahore 54000, Pakistan; 2 Acromed Invest AB, Magistratsvägen 10, Lund SE-22643, Sweden; E-Mail: bd@acromed.se; 3 Department of Science and Technology, Campus Norrköping, Linköping University, Norrköping SE-60174, Sweden; E-Mail: magnus.willander@liu.se

**Keywords:** ZnO nanowire/nanorods, functionalization, intracellular measurement, glucose, metal ions, human fat cells, frog oocytes, electrochemical sensor

## Abstract

Recently ZnO has attracted much interest because of its usefulness for intracellular measurements of biochemical species by using its semiconducting, electrochemical, catalytic properties and for being biosafe and biocompatible. ZnO thus has a wide range of applications in optoelectronics, intracellular nanosensors, transducers, energy conversion and medical sciences. This review relates specifically to intracellular electrochemical (glucose and free metal ion) biosensors based on functionalized zinc oxide nanowires/nanorods. For intracellular measurements, the ZnO nanowires/nanorods were grown on the tip of a borosilicate glass capillary (0.7 µm in diameter) and functionalized with membranes or enzymes to produce intracellular selective metal ion or glucose sensors. Successful intracellular measurements were carried out using ZnO nanowires/nanorods grown on small tips for glucose and free metal ions using two types of cells, human fat cells and frog oocytes. The sensors in this study were used to detect real-time changes of metal ions and glucose across human fat cells and frog cells using changes in the electrochemical potential at the interface of the intracellular micro-environment. Such devices are helpful in explaining various intracellular processes involving ions and glucose.

## 1. Introduction

Nanotechnology and nanoscience offer new ways of manufacturing intracellular medical devices based on bioactive nanoscale structures. This can bring fundamental changes to the measurements and understanding of biological processes in health and disease, as well as enable novel diagnostics and interventions for treating diseases like diabetes [1,2]. The size domains of the components involved with nanotechnology are similar to those of biological structures. For example, the diameter of nanowires/nanorods is about the same size as for a protein (<10 nm). Because of this similarity in scale and certain functional properties, nanotechnology is a natural succession of intracellular research such as on semiconductor metal oxides, synthetic and hybrid nanostructures that can sense and repair biological lesions, damages and diagnostics and interventions for treating diseases like diabetes [2]. Recently nanotechnology has enabled intracellular sensing technologies that provided more accurate medical information for diagnosing diseases more rapidly, and miniature devices that could manage treatment automatically [2,3,4,5]. Nanotechnology has introduced new and exciting opportunities by using novel intracellular techniques based on nanostructure materials, for instance glucose biosensors [3]. Intracellular biosensor analysis of biochemical species has stimulated a clearly dominating interest due to the importance of intracellular measurement for control of different diseases. In this review we demonstrate a functionalized ZnO nanostructure-based electrochemical sensor for selective detection of intracellular bio-chemical species. Hexagonal ZnO nanowires/nanorods were grown on the tip of a silver-covered borosilicate glass capillary (diameter 0.7 µm) to make possible microinjection of specific reagents, which can interrupt or activate signal transmission from analytes, into the relatively large cells of adipocytes and oocytes [3,4,5].

## 2. ZnO Nanostructures

Zinc oxide (ZnO) is a group II-VI semiconductor wide band gap material with a band gap energy of 3.37 eV at room temperature and a large excitonic binding energy of 60 MeV. It is a biosafe and biocompatible material. Zinc oxide is a polar semiconductor with two crystallographic planes with opposite polarity and different surface relaxation energies. This leads to a higher growth rate along the c-axis. The crystal structures formed by ZnO are wurtzite, zinc blende, and rocksalt. ZnO is an important multifunctional material that has wide applications in chemical, and biochemical sensors and optical devices [6]. In this review ZnO nanorods will be discussed in the context of electrochemical biosensors for intracellular measurements.

The synthesis of ZnO nanostructures has been an active field for the last fifteen years because of their wide applications as biosensors, transducers and catalysts. In recent years, semiconducting nanostructures have been the focus of considerable research due to their unique properties that can be exploited in various functional nano-devices especially for intracellular sensors [3,4,5,7]. Nano-device functionality has been demonstrated with these nanostructure materials in the form of electric field-effect switching [8], single electron transistors [9], biological and chemical sensing [10], and luminescence [11] for one dimensional semiconducting nanostructures. 

Due to the small dimensions of nanowires/nanorods combined with a very large contact surface and strong binding with biological and chemical reagents, nanowires/nanorods will have important applications for intracellular environment. The diameter of these nanostructures would be comparable to the size of the biological and chemical species being sensed, which intuitively makes them represent excellent primary transducers for producing electrical signals. A literature survey reveals that ZnO nanorods show n-type semiconducting property and their electrical transport is highly dependent on the adsorption/desorption nature of chemical species [12]. Among a variety of nano-sensor systems, our nanostructure electrochemical probe for intracellular measurement is one that can offer high sensitivity and real-time detection. 

## 3. ZnO Nanostructure Growth and Characherisations

**Figure 1:**
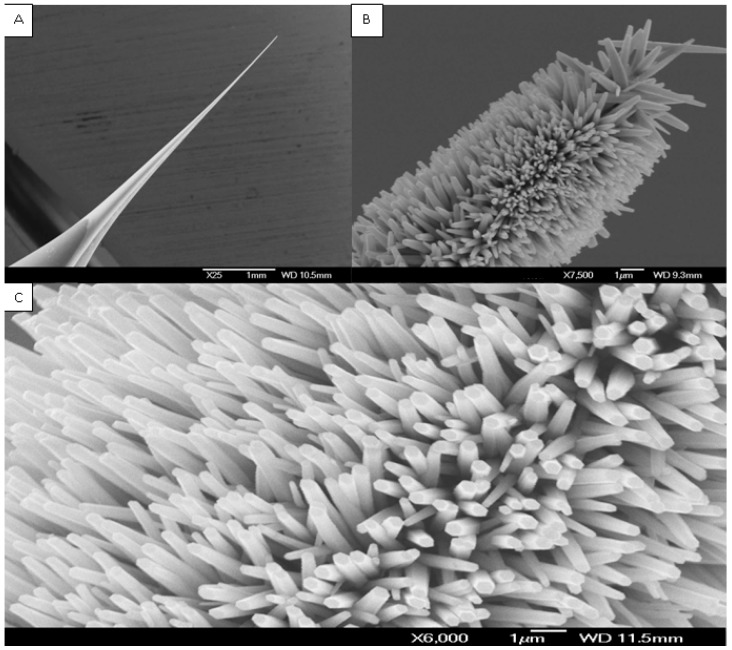
Field emission scanning electron microscope images at different magnifications of the Ag-coated glass tip without (**A**) and with (**B**,**C**) grown ZnO nanorods [3,5].

For intracellular measurement, ZnO nanostructures were grown on the tip of borosilicate glass capillaries using an aqueous chemical growth (ACG) technique. Equal molar concentrations of two solutions, zinc nitrate hexahydrate [Zn(NO_3_)_2_·6H_2_O, 99.9% purity] and hexamethylenetetramine (C_6_H_12_N_4_, 99.9% purity) were used for growth of ZnO nanowires/nanorods on the glass tip. The glass capillary substrates were immersed into the solution and tilted against the wall of the beaker that, was put into an oven at low temperature for different times to get aligned ZnO nanostructure [3,4,5]. Structural morphology and size distribution of the ZnO nanowires/nanorods were investigated by Field Emission Scanning Electron Microscope (FESEM, JSM-6335F Scanning Electron Microscope, JEOL, Norrköping, Sweden) at different magnifications. The ZnO nanowires/nanorods cover a small part of the silver-coated glass tip varying from 3 mm and down to 10 µm. The sensing electrode with ZnO hexagonal nanorods is shown in Figure 1. The nanostructure has a rodlike shape with a hexagonal cross section and primarily aligned along the perpendicular direction. The nanorods are uniform in size with a diameter of 100–120 nm and a length of 900–1000 nm. 

## 4. ZnO Nanostructure-Based Intracellular Measurements

### 4.1. Intracellular Glucose Measurements

Glucose is one of the most important studied analytes due to its immense importance in the human body, medicine, the environment and the food industry. Electrochemical intracellular biosensors have been of great interest during the last few years for the accurate detection of glucose concentrations. Electrochemical biosensors based on ZnO nanowires/nanorods (potentiometric, amperometric, impedometric and conductometric) are widely employed for intra/extracellular glucose sensing [3,7,13]. Glucose is a fundamental carbohydrate in biology and glucose is one of the main products of photosynthesis and the human body’s primary source of energy. The living cell uses it both as a source of energy and as a metabolic intermediate in the synthesis of fats, for instance. When glucose levels in the bloodstream are not properly regulated, diseases such as diabetes could develop. Because of the high demand for blood-glucose monitoring, significant research has been devoted to produce reliable methods for *in vitro* or *in vivo* glucose measurement. Accurate and rapid measurement of glucose is obviously critical for diagnosis and treatment of the different forms of diabetes, as well as in research to understand the diabetes diseases and to develop new therapies. It is desirable to be able to get continuous measurements not only of circulating glucose, but also of interstitial and of intracellular concentrations in the living human being and in living cells, respectively. There are not many intracellular biosensors reported in the literature. A glucose micro-sensor with a tip diameter of 2 µm that could provide reliable and accurate measurements of glucose during few hours has been described [14]. As an alternative to direct measurements in cells, metabolite concentrations can be assessed by indirect, non-invasive measurements using various techniques, such as NMR that gives average concentrations on samples containing several cells [15]. Other techniques, like those employing fluorescently labeled glucose molecules, may permit real time measurements in single living cells [16]. A precise and fast measurement of glucose is obviously significant to diagnosis and handling of the different forms of diabetes, as well as in research to understand the diabetes diseases and to develop new therapies [3,4,5]. In recent years the research community has been working to miniaturize the materials for intracellular measurements in biological and biomedical applications. There has been great progress in the application of metal-oxide nanomaterials such as ZnO and CuO, in intracellular biosensors [3,4,5,7,17,18,19].The continuous progress in synthesizing and fabricating controlled materials on the submicron and nanometer scale results from novel advanced functional materials with tailored properties. When bulk materials are scaled down to nanoscale, most of them reveal novel properties that cannot be deduced from their bulk behavior. For the fabrication of an efficient and fast biosensor, the choice of substrate for dispersing the sensing material decides the sensor performance. In a previous investigation we measured concentrations of intracellular glucose concentrations using ZnO nanorods [3,7]. Intracellular determination of glucose was of great interest and ZnO nanowire/nanorod technology has potential for such measurements. The focus of the current review is the demonstration of a ZnO nanorods-based sensor suitable for intracellular selective glucose measurements and metal ion detection. The main efforts were given to the construction of tips selective for glucose and capable of penetrating the cell membrane, as well as the optimization of the electrochemical potential properties. Tips of borosilicate glass capillaries (0.7 µm in diameter) with grown ZnO nanorods (Figure 1) have proven to be a convenient and practical choice as we have demonstrated before with our intracellular Ca^2+^ and pH nano-sensors developed earlier [4,5].

Enzymes are biological recognition molecules commonly used in research and development because most chemical reactions in living systems are catalyzed by very specific enzymes. As a consequence, immobilization strategies for enzymes are important to preserve their biological activity [3]. Glucose oxidase is widely employed in glucose biosensors due to its stability and high selectivity for glucose [7]. In the study reported here glucose oxidase was immobilized directly on the ZnO by electrostatic interaction.

**Figure 2:**
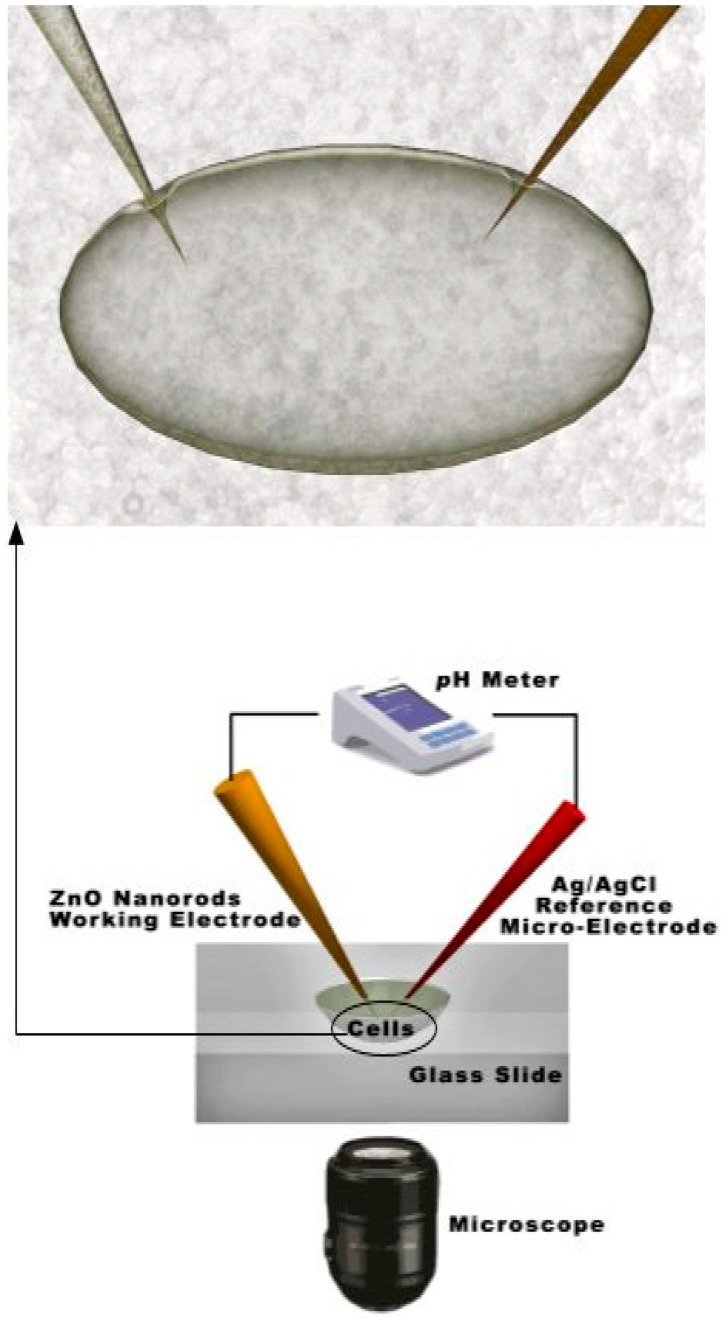
Schematic experimental setup for the intracellular potentiometric measurements.

The determination of the intracellular glucose concentration with functionalized ZnO nanorod-coated microelectrodes was performed in two types of cells, human adipocytes and frog oocytes [3,7]. Schematic diagram for intracellular measurements is shown in Figure 2.

**Figure 3:**
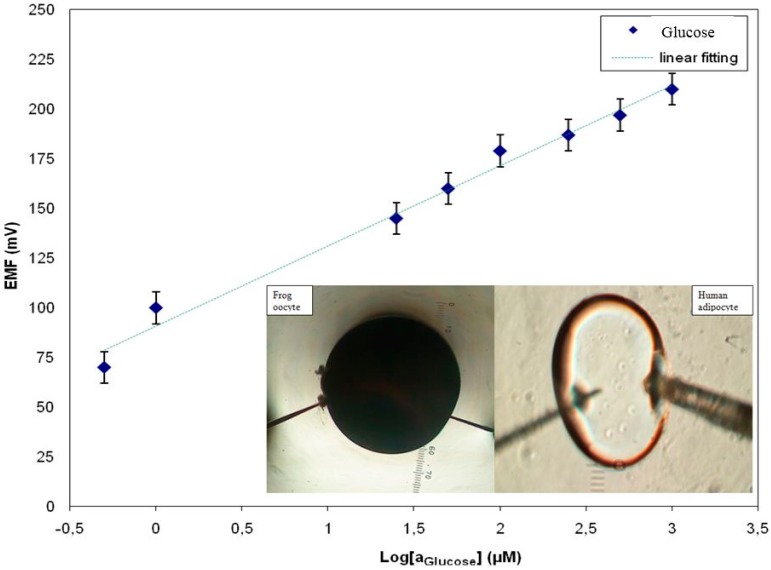
A calibration curve showing the electrochemical potential difference *vs.* the Ag/AgCl reference electrode in response to the glucose concentration using the functionalized ZnO nanorods as working electrode [3].

In the human body, the hormone insulin only stimulates glucose transport into muscle and fat cells. However, insulin has also been found to affect glucose uptake in oocytes from the frog *Xenopus laevis* [20,21]. The large size of these cells makes it possible to microinject specific reagents that interrupt or activate signal transmission to glucose. A dynamic range of glucose concentrations was made in 10 mM Phosphate Buffered Saline (PBS) containing 1.5 mM Na_2_HPO_4_, 48 mM KH_2_PO_4_, 0.135 mM sodium chloride and 2.7 mM KCl at pH 7.4. The response of the electrochemical potential difference of the ZnO nanorods *vs.* reference electrode to the changes in buffer electrolyte enabled glucose measurements the range of 500 nM to 1 mM and shows that the glucose dependence is linear with sensitivity equal to 42.5 mV/decade (Figure 3) [3,7]. The intracellular glucose concentration in the human adipocytes was 50 ± 15 µM (*n* = 5), which can be compared with the 70 µM intracellular concentration determined by nuclear magnetic resonance spectroscopy in rat muscle tissue in the presence of a high, 10 mM, extracellular glucose concentration [15]. For the frog oocytes, it was 125 ± 23 µM (*n* = 5). When we achieved a stable potential for the intracellular measurement, 10 nM insulin was added to the extracellular solution. After few minutes, the insulin increased the glucose concentration in the human adipocyte from 50 ± 15 to 125 ± 15 µM and the glucose concentration in the frog oocytes increased from 125 ± 23 µM to 250 ± 19 µM [3]. The reported values of the glucose concentration in human adipocytes and frog oocytes using our functionalized ZnO nanorods sensor were consistent with values of glucose concentration reported in the literature survey [3].

### 4.2. Intracellular Metal Ion Measurements

Metal ions play dominating roles in medical physics, especially for cell morphology, so it is important to detect the metal ion concentration and changes in concentration of specific ions for cell biology. The most prominent ions are Ca^2+^, Mg^2+^, K^+^, Na^+^, Fe^2+^, Cu^2+^, Zn^2+^, *etc.* Every ion has its own chemistry and fundamental biology with different physical and chemical characteristics [1]. All the metal ions play important roles in biological systems. Every metal ion has its own importance in living systems by acting as cofactors in enzymes, as osmotic regulators, as current carriers and consequently as factors in information processing and as integrator and stabilizers of proteins and lipids [22,23]. Recently, nanostructured ZnO-based ion-selective sensors were fabricated by effective, simple techniques for measuring specific ion concentrations in intracellular as well as extracellular environments. Table 1 is a summary of the intracellular sensors studied.

**Table 1:** Summary of ZnO nanostructure-based intracellular metal ion-selective biosensors (PVC: Poly Vinyl Chloride; BGC: Borosilicate Glass Capillary).

Ref	[24]	[5]	[25]	[18]	[17]	[26]
**Enzyme/Electrode**	PVC/Ag wire	PVC/BGC	PVC/Ag wire	PVC/IBGC	PVC/BGC	PVC/BGC
**Immobilization Mode**	Physical adsorption	Physical adsorption	Physical adsorption	Physical adsorption	Physical adsorption	Physical adsorption
**ZnO Synthesis**	ACG	ACG	ACG	ACG	ACG	ACG
**ZnO Morphology**	Nanowires	Nanorods	Nanorods	Nanorods	Nanorods	Nanorods
**Response Time(s)**	<1 min	fast	20 s	fast	fast	fast
**Ion-Selection**	Ca^2+^	Ca^2+^	Ca^2+^	Na^+^	Mg^2+^	K^+^
**Sensitivity (mV/decade)**	26.4	29.67	113.92	72	26.1	41.47
**Linaer Response Range**	1 µM–0.1 M	100 nM–10 mM	1 µM–1 mM	0.5 mM–100 mM	500 nM–100 mM	25 µM–125 mM
**Interfering Ions**	No tested	No tested	Na^+^, Mg^2+^, K^+^	Ca^2+^, K^+^, Mg^2+^,	Ca^2+^, Na^+^, K^+^	No tested

Calcium ions play important roles in regulating enzyme activity, neuronal activity, muscle contraction, vesicle exocytosis, cell development and death [5,24]. Clinical situations in which the *in vivo* monitoring of Ca^2+^ is of interest include, for example, organ-transplantations, hemodialysis or exchange transfusion, during which rapid change in the concentration of the ionized calcium may occur, therefore it is important to know its concentration in different types of extra- and intracellular compartments [5,24].Such applications have made Ca^2+^ one of the most interesting elements to sense. Other industrial applications are Ca^2+^ ion measurements in boiler water, soils and fertilizers. 

### 4.3. Intracellular Calcium Ion Measurements

In our previous investigations we have successfully measured concentrations of intracellular Ca^2+^ using ZnO nanorods [5]. Intracellular determination of Ca^2+^ was of great interest and ZnO nanorod technology has potential for such measurements. The focus of the current study was the demonstration of a ZnO nanorods-based sensor suitable for intracellular selective Ca^2+^ detection. Our main effort has been directed towards the construction of tips selective for Ca^2+^capable of penetrating the cell membrane as well as the optimization of the electrochemical potential properties (Figure 2).

Tips of borosilicate glass capillaries (0.7 µm in diameter) with grown ZnO nanorods have proven to be a convenient and practical choice as we have demonstrated before with our earlier developed intracellular nanosensors for glucose, metal ions and pH [1,3,4,5,7,17,18,19].

The ZnO nanorods coated with ionophore-containing membranes were highly sensitive to detect and monitor Ca^2+^ with capability for *in vivo* measurements during biological processes [5].

The selective intracellular measurement methods utilizes two electrodes: (1) A ZnO nanorod-decorated electrode coated with ionophore-containing membrane as the intracellular working electrode and (2) an Ag/AgCl electrode as the intracellular reference microelectrode. The electrochemical potential difference response recorded in this way measures the difference in electrochemical surface potential generated near the electrodes. The nanorods are all grown on the same conducting surface and will have the same potential [5].

ZnO nanowire/nanorods were grown as hexagonal single ZnO nanowire/nanorod crystals using a low-temperature method on asilver-coated capillary glass tip [5]. This is shown in Figure 1. The ZnO nanowire/nanorod covers a small part of the silver-coated film. The part of the capillaries covered with ZnO nanowires/nanorods varied from 3 mm and down to 10 µm. The sensing electrode with ZnO hexagonal nanorods is shown in Figure 1. The nanostructure has a rodlike shape with a hexagonal cross section and is primarily aligned along the perpendicular direction. The nanorods are uniform in size with a diameter of 100–120 nm and a length of 900–1000 nm. The electrical contacts are made on the other end of the Ag film. The ZnO nanorod layer on the silver-coated capillary glass tip was coated with an ionophore membrane to make it most selective for specific metal ions by a manual procedure.

The electrochemical potential of all metal ions-probe were measured with a model 827 pH meter (Metrohm, Herisau, Switzerland) *vs.* an Ag/AgCl micro-reference electrode. Calibration curves show an approximately stable potential difference against each concentration of Ca^2+^ range of 100 nM to 10 mM. Our results showed a good improvement in electrode stability by using Ag/AgCl as reference microelectrodes. After making the calibration curve with metal ion samples in buffer solutions, the probe was used to selectively measure the ionic concentration in two types of cells; these were human adipocytes (fat cells) and frog oocytes (egg cells). The experimental setup for the intracellular measurements is shown in Figure 2.

Human adipocytes (fat cells) were isolated by collagenase digestion of pieces of subcutaneous adipose tissue [27] obtained during elective surgery at the university hospital in Linköping, Sweden. Cells were incubated for a few hours before use as described in [28]. A glass slide substrate (5 cm length, 4 cm width, and 0.17 mm thickness) with sparsely distributed fat cells was placed on the prewarmed microscope stage set at 37 °C. 

Female *Xenopuslaevis* frogs were anesthetized in a bath with tricaine (1.4 g/L, Sigma-Aldrich, Linköping, Sweden) and the ovarian lobes cut off through a small abdominal incision (a procedure approved by the local Animal Care and Use Committee at Linköping University). Oocytes were manually dissected into smaller groups and defolliculated by enzymatic treatment with liberase (Roche Diagnostics, Linköping, Sweden) for 2.5 h. Stage V and VI oocytes (approximately 1 mm in diameter) without spots and with clear delimitation between the animal and vegetal pole were selected. Oocytes were kept in MBS solution (88 mM NaCl, 1 mM KCl, 2.4 mM NaHCO_3_, 15 mM HEPES, 0.33 mM Ca(NO_3_)_2_, 0.41 mM CaCl_2_, 0.82 mM MgSO_4_, 2.5 mM pyruvate, 25 mg/L penicillin-streptomycin; all from Sigma-Aldrich, at 11 °C for 1–5 days before measurements. During measurements, oocytes were placed in a Perspex chamber and bathed in a control solution (in mM: 88 NaCl, 1 KCl, 15 HEPES, 0.4 CaCl_2_, and 0.8 MgCl_2_. NaOH was used to set pH to 7.4 yielding a final sodium concentration of ~100 mM). The experimental procedures are described in more detail by [29]. Measurements were carried out at room temperature (20–23 °C). The indicator electrode and reference electrode were mounted and micromanipulated into the oocytes according to the procedure described for adipocytes [4]. 

**Figure 4:**
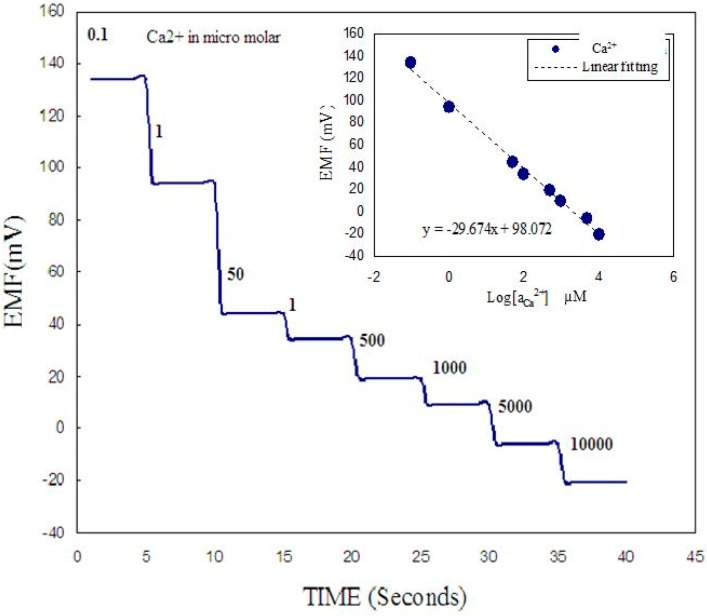
Shows potentiometric response *vs.* time as the concentrations of Ca^2+^ concentration is changed in the buffer surrounding the cell for the case where for partial insertion of the functionalized ZnO nanorods. The insert shows a typical the calibration curve of the present working electrode [5].

A two-electrode configuration was used for electrochemical studies consisting of ZnO nanorod metal ion such as Ca^2+^ sensor as the working electrode and Ag/AgCl as a reference microelectrode. The experimental setup for the intracellular measurements is shown in Figure 2. The response of the electrochemical potential difference of the ZnO nanorods to the changes in buffer electrolyte Ca^2+^ was measured for a range of 100 nM to 10 mM and shows that this Ca^2+^ dependence is linear and has good sensitivity. This linear dependence implies that such sensor configuration can provide a large dynamic range as shown in Figure 4. After calibrating our ZnO base Ca^2+^ sensor, we used the nano-sensor to measure the free concentration of intracellular Ca^2+^ in a single human adipocyte. The Ca^2+^-selective nano-electrode, mounted on a micromanipulator, was moved into position in the same plane as the cells. The ZnO nanoelectrode and the reference microelectrode were then gently micromanipulated a short way into the cell (Figure 2) and signal was detected to identify the activity of calcium ions. After interpolation of the results with the calibration curve, the intracellular Ca^2+^ concentration was 123 ± 23 nM. The measured intracellular concentration was closeto the intracellular concentration determined after loading human adipocytes with the Ca^2+^-binding fluorophore fura-2 [30]. In a second experiment we used the nano-sensor to measure intracellular Ca^2+^ concentration in single frog oocytes. The intracellular Ca^2+^ concentration was 250 ± 50 nM, which was also close to the value of a previous report [31].

**Figure 5:**
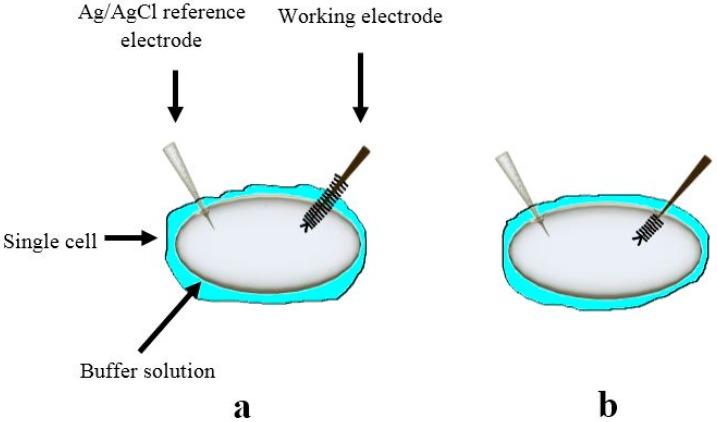
Schematic diagram showing the principle of measurements with different penetration depths during the experiment (**a**) the case with partial insertion of the functionalized ZnO nanorods and in (**b**) when all the functionalized ZnO nanorods are inserted inside the cell [5].

To test the sensitivity of our constructed biosensor we performed measurements in two cases, first while changing the Ca^2+^ concentration in the buffer solution around the cell where half of the working functionalized ZnO nanorods were inserted inside the cell and the other half was in contact with the surrounding buffer solution (Figure 5a). The Ca^2+^ was varied from 100 nM to 10 mM. The second configuration was when all the functionalized ZnO nanorods were inserted inside the cell (Figure 5b). Significantly, as illustrated in Figure 4, the functionalized ZnO nanorods exhibited a stepwise decrease in the induced electrochemical potential only in the case of the configuration shown Figure 5a. Once the functionalized ZnO nanorod working electrode was totally inserted inside the cell (Figure 5b) the electrochemical potential difference signal detected was stable even when the Ca^2+^ concentration was varied in the buffer solution. This implies that the constructed working electrode is sensing with good sensitivity and stability. In addition, this observation confirms that the values of the potentiometric response when the whole sensing electrode was inserted inside the cell membrane is the signal corresponding to the Ca^2+^ concentration value inside the cell. The inset in Figure 4 shows the calibration curve of the present constructed biosensor [5].

**Figure 6:**
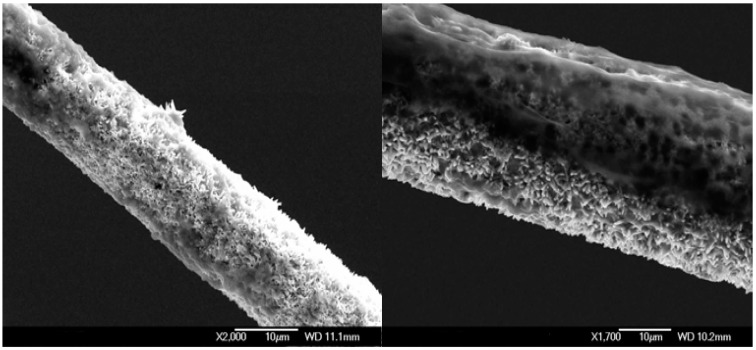
Scanning electron microscopy images showing the working electrode after intracellular measurements at two different magnifications.

The viability of the penetrated cells depends strongly on the size of the ZnO nanorods. By reducing the length of the ZnO nanorods, the total diameter of the tip will be reduced, which in turn increases the cell viability and the sensitivity of the device is also expected to increase. When the functionalized working electrode was removed outside the cells after measurements, it was monitored by microscopy (Figure 6). Figure 6 shows different magnification SEM images from the working electrode after measurements. The ZnO nanorods were not dissolved. This result was expected because the functionalization provided protection for the surface of the nanorods [5].

### 4.4. Intracellular Magnesium Ion Measurements

The magnesium ion (Mg^2+^) is an important intracellular divalent cation in living cells, where it plays a major biological function in its participation with phosphate compounds and phosphate metabolism, binding in chlorophyll pigments, and acting as an important cofactor in numerous enzymatic reactions [17,22]. Mg^2+^also influences the nervous impulse, tension growth in muscle, and modulates amongst others the ionic transport in nerve and mitochondria [17,23]. There was increased demand for selective, sensitive, and fast chemical sensors to explore the physiological environment. In particular, concentrations of Mg^2+^ in the intracellular compartments are difficult to measure. Here we describe a new Mg^2+^-selective probe to measure intracellular Mg^2+^ concentration. The development of miniaturized ion-selective electrodes has received extensive attention during the recent years and these microelectrodes are useful for intracellular measurement as we have demonstrated in previous investigations [3,4,5,17].

**Figure 7:**
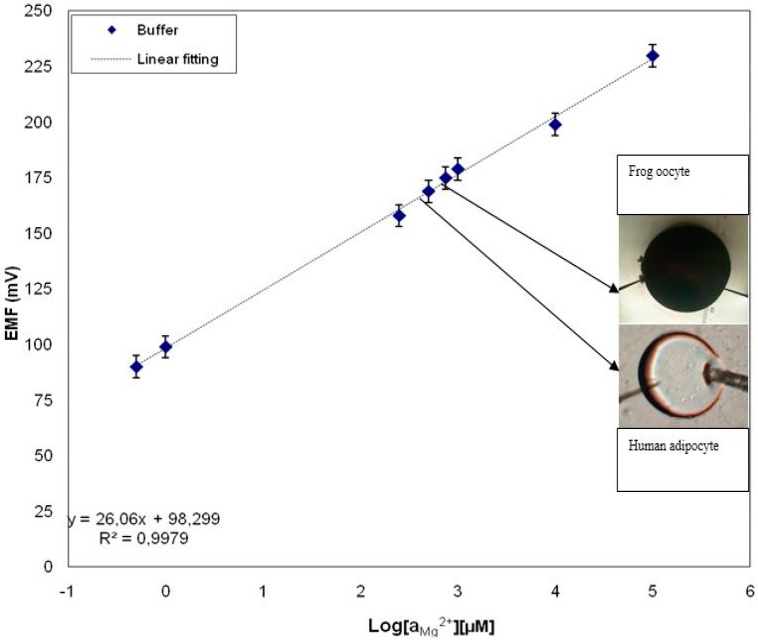
A calibration curve showing the electrochemical potential difference between the Mg^2+^-selective ZnO nanorod-covered and the Ag/AgCl reference microelectrode *vs.* the Mg^2+^ concentration. Insets show images of human adipocytes and frog oocytes with arrows pointing at measured intracellular levels of Mg^2+^ for the respective cells [17].

**Figure 8:**
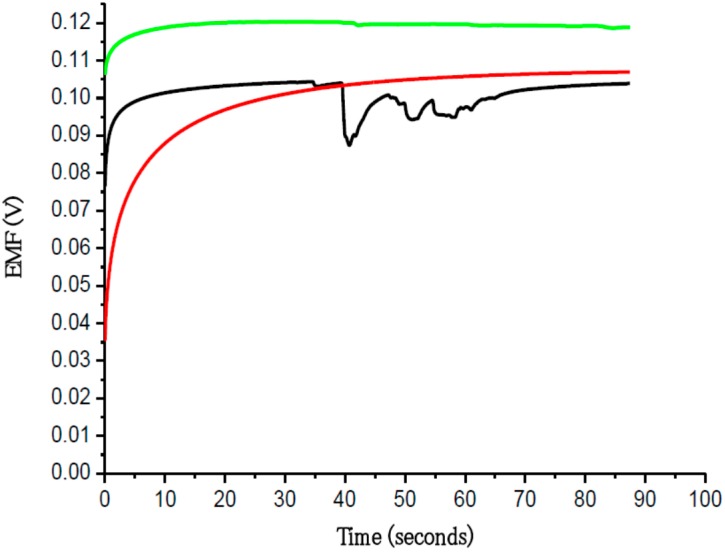
The output response with (black and green) and without (red) interfering ions [17].

Functionalized ZnO nanorods were used for intracellular quantification of magnesium ions in the same way we have measured other metal ions, such as intracellular calcium. For magnesium the ZnO nanorods were functionalized by covering them with a Mg^2+^-selective membrane. The potential difference between the Mg^2+^-selective probe and the reference microelectrode was found to be linear over a wide concentration range of Mg^2+^ (500 nM to 100 mM) (Figure 7). We measured the free concentration of intracellular Mg^2+^ in single human adipocytes and frog oocytes by micromanipulating the Mg^2+^-selective and the reference microelectrodes gently into the cells (Figure 2). The measured intracellular Mg^2+^ concentration for human adipocytes was 0.4 to 0.5 mM and for frog oocyte it was 0.8 to 0.9 mM, Asif *et al.* [17] closely consistent with the intracellular concentration reported in [17]. Selectivity is the most important characteristic which describes the specificity towards the target ion in the presence of other ions (interfering ions). There are a number of different methods to check the potentiometric selectivity [17]. We checked the selectivity and stability of the sensor by output response curve with (black and green) and without (red) interfering ions as shown in Figure 8. The red curve shows the output response without any interfering ions in 1 µM concentration of Mg^2+^ showing good stability after reaching at stable point. The black curve shows that as soon as we introduced the interfering ions in higher concentration of other metal ions such as Ca^2+^, Na^+^, K^+^ in a 1 µM MgCl_2_ solution, disturbed the stability for a short time, but after these initial disturbances the signal became stable and reached the same value as before the introduction of the interfering ions. The green curve shows the output response with same amount of interfering ions. Here only small peaks were observed during the measurements [17].

### 4.5. Intracellular Sodium and Potassium Ion measurements

Sodium ions, Na^+^ and potassium ions K^+^ are abundant in the extracellular space and have important roles in the excitability of nerve and muscle cells, and in water and salt homeostasis in biological systems [32,33,34]. While the intracellular concentration of Na^+^ is typically low in such environments, and the role of intracellular Na^+^ has not been fully understood, the intracellular concentration of K^+^ was relatively higher compared to that of Na^+^. However, during the last decade several studies have revealed that Na^+^ may act as a second messenger in different cell types. Na^+^ regulates different transporters and receptors in renal cells and neurons, and it regulates the activation of both Na^+^ and K^+^ channels, thereby regulating the re-absorption of salt and water in the kidneys and neuronal excitability [18,19]. Na^+^-channels and the intracellular concentration of Na^+^ are important in the early stages of apoptotic processes in cell lines, and Na^+^ may be involved in intracellular signaling during apoptosis [16]. Potassium is the main intracellular ion in the body and its levels are crucial to normal homeostasis. It is mainly contained within the intracellular fluid (ICF) compartment, with only about 2% of the total body potassium residing in the extracellular fluid (ECF). Here, we describe the application of ZnO nanorods inside cells. The use of ZnO nanorods for intracellular detection of biological analytes, metallic ions, and clusters has been developed by our group. We have performed preliminary intracellular detection of most of the basic metallic ions, and glucose in oocytes and adipocyte cells [1,3,5,7,17,18,19]. The objective of this review is to characterize ZnO nanorods and present their application as an intracellular potentiometric selective ion sensor. Intracellular determination of Na^+^ and K^+^ was of great interest and ZnO nanorod technology has the potential for such measurements. We demonstrate a ZnO nanorod-based sensor suitable for intracellular selective Na^+^ and K^+^ detection as well as the optimization of its electrochemical properties. This sensor is based on ZnO nanorods grown on a Borosilicate glass capillary that is capable of penetrating a cell membrane.

**Figure 9:**
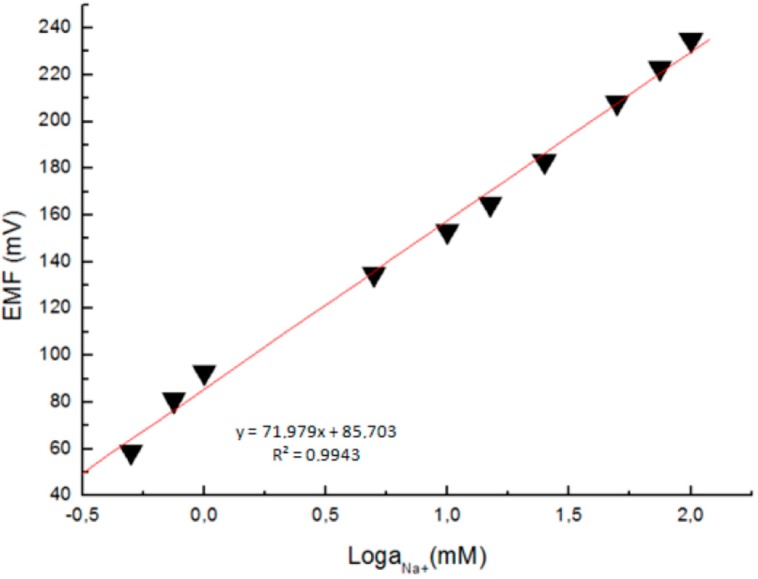
A calibration curve showing the electrochemical potential difference between the Na^+^-selective ZnO nanorod and the Ag/AgCl reference microelectrodes *vs.* the Na^+^ concentration [18].

The response of the electrochemical potential difference of the ZnO nanorods to the changes in buffer electrolyte Na^+^ was measured with a range from 0.5 mM to 100 mM. This demonstrates that the Na^+^ dependence is linear and has sensitivity of to 72 mV/decade at around 23 °C (Figure 9). This linear dependence implies that such sensor configuration can provide a large dynamical range [18].

The sensor showed agood performance in sensitivity, stability, selectivity, reproducibility for Na^+^ detection and small interference from other ions. Furthermore, the sensor is easy to fabricate and easy to insert in large cells. The measured intracellular Na^+^ and K^+^ concentrations in single human adipocytes and frog oocytes were consistent with values found in the literature. These results pave the way to perform biologically relevant measurements of Na^+^ and K^+^ detection inside living [18,19].

**Figure 10:**
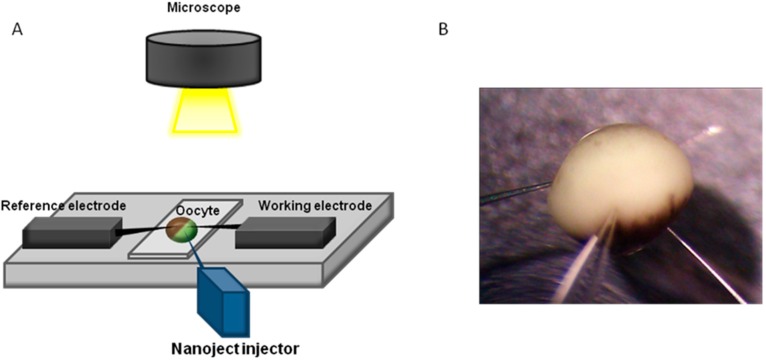
Experimental setup for simultaneous test solution injection and potentiometric measurements, (**A**) Schematic illustration of the setup and (**B**) Photography of Xenopus oocyte penetrated by the reference electrode (left), measurement electrode (right), and injector (middle).

After the success of the intracellular metal ions detection, intracellular physiological change of metal ions such as K^+^ alteration measurement by using ZnO nanostructure based microelectrode parallel with electrophysiological verification was of great interest. The aim of this work was therefore to study sensitivity and the possibility of using the K^+^ specific microprobe to detect changes in intracellular K^+^ concentrations in xenopus oocytes following injection of various test solutions. Potentiometric measurements were done in parallel with electrophysiological measurements to verify the accuracy of the detected concentrations [26]. The experimental setup for injection and detection of K^+^ is shown in Figure 10.

**Figure 11:**
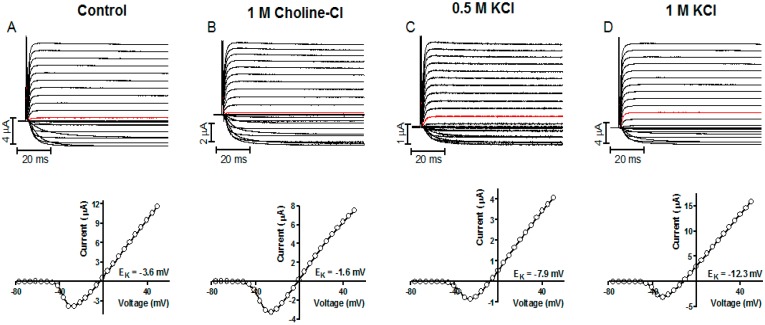
Representative K^+^ current recordings and the corresponding I(V) curves measured electrophysiological in Kv channel expressing *Xenopus* oocytes. (**A**) Shows data for control oocytes and (**B**–**D**) for oocytes injected with indicated test solution. The holding potential was set to −80 mV and test pulses ranging from −80 to + 50 mV. The current generated by stepping to 0 mV is marked in red in each recording [26].

To evaluate the accuracy of potentiometric data, the intracellular K^+^ concentration was measured with both electrophysiological and potentiometric methods on the same oocyte. Two-electrode voltage clamp measurements of K^+^ currents were performed as previously described [29]. The holding potential was set to −80 mV and the currents achieved by stepping to potentials between −80 and +50 mV for 100 ms in 5 mV increments. The amplifier capacitance and leakage compensation was used. Each oocyte was then immediately transferred for potentiometric measurements (oocytes bathed in 100 mM KCl solution).ll oocytes were measured both electrophysiologically and potentiometrically within 25 min after test solution injection. The [K^+^]i was tested electrophysiologically to be stable for at least 25 min after 1M KCl injection [26]. The concentration determined with the two methods gave almost the same results (Figure 11).

**Figure 12:**
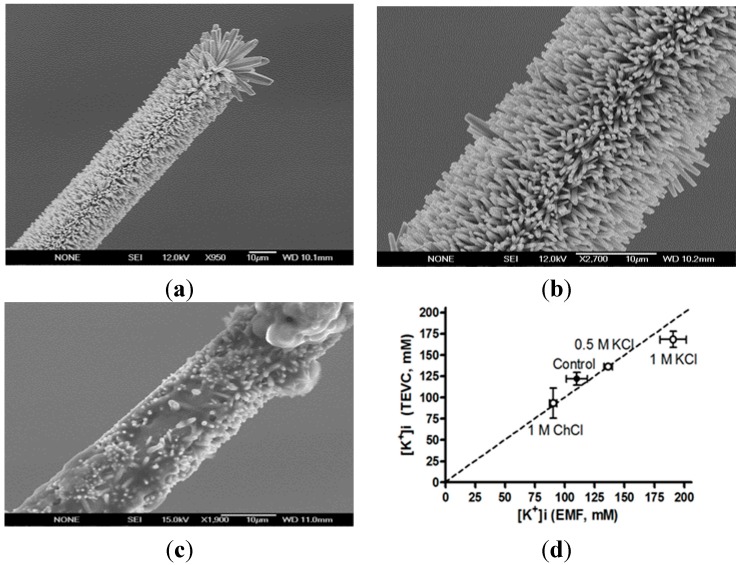
Intracellular K^+^ concentrations in Kv channel-expressing *Xenopus*oocytes measured with electrophysiological and K^+^-selective microelectrode methods. The Field emission scanning electron microscopy images of the K^+^-selective microelectrode before (**a**,**b**) and after intracellular measurements (**c**). Data points are expressed as mean values for control oocytes and oocytes injected with 50 nL of indicated test solution (**d**). Error bars show SE. *n* = 3–5) [26].

All the microelectrodes were investigated pre- and post-experimentally with scanning electron microscopy to make sure that the nanorods on the K^+^-selective microelectrodes were not dissolved (Figure 12).

## 5. Conclusions

Hexagonal Zn Onanowires/nanorods successfully were grown on the tip of a borosilicate glass capillary (diameter 0.7 µm) to make possible microinjection of specific reagents, which can interrupt or activate signal transmission from analytes, into the relatively large cells of adipocytes and oocytes. The as grown ZnO nanowires/nanorods were functionalized for intracellular selective glucose and metal ions measurements in single human adipocytes and frog oocytes. This is a technique offering new approaches in the investigation of glucose and metal ions for intracellular measurements. The proposed intracellular nano-biosensor showed a fast response with a time constant of less than 1 s and had a wide linear range. The performance regarding sensitivity, selectivity, and freedom from interference when the sensor was exposed to intra- and extracellular glucose and metal ions measurements were quite acceptable. The stability of the sensing ZnO layer was, however, limited and should be improved although the experiments described here have a short duration and could be performed without influence of this drawback. These results demonstrate the capability to perform biologically relevant measurements of glucose and metal ions within living cells. After the success of the intracellular metal ions detection, intracellular physiological change of metal ions such as K^+^ alteration measurement by using ZnO nanostructure based microelectrode parallel with electrophysiological verification was of great interest. The aim of this work was therefore to study sensitivity and the possibility of using the K^+^ specific microprobe to detect changes in intracellular K^+^ concentrations in xenopus oocytes following injection of various test solutions. Potentiometric measurements were done in parallel with electrophysiological measurements to verify the accuracy of the detected concentrations. The ZnO-nanorod electrode thus holds promise for minimally invasive dynamic analyses of single cells. All of these advantageous features can make the proposed nano-biosensor applicable in medical, food or and other areas. 
